# Research Status in the Use of Surface-Enhanced Raman Scattering (SERS) to Detect Pesticide Residues in Foods and Plant-Derived Chinese Herbal Medicines

**DOI:** 10.1155/2024/5531430

**Published:** 2024-01-12

**Authors:** Bing-Yan Chu, Chi Lin, Peng-Cheng Nie, Zheng-Yan Xia

**Affiliations:** ^1^School of Pharmacy, Zhejiang University of Technology, Hangzhou 310014, China; ^2^School of Medicine, Hangzhou City University, Hangzhou 310015, China; ^3^College of Biosystems Engineering and Food Science, Zhejiang University, Hangzhou 310058, China

## Abstract

Surface-enhanced Raman scattering (SERS) technology has unique advantages in the rapid detection of pesticides in plant-derived foods, leading to reduced detection limits and increased accuracy. Plant-derived Chinese herbal medicines have similar sources to plant-derived foods; however, due to the rough surfaces and complex compositions of herbal medicines, the detection of pesticide residues in this context continues to rely heavily on traditional methods, which are time consuming and laborious and are unable to meet market demands for portability. The application of flexible nanomaterials and SERS technology in this realm would allow rapid and accurate detection in a portable format. Therefore, in this review, we summarize the underlying principles and characteristics of SERS technology, with particular focus on applications of SERS for the analysis of pesticide residues in agricultural products. This paper summarizes recent research progress in the field from three main directions: sample pretreatment, SERS substrates, and data processing. The prospects and limitations of SERS technology are also discussed, in order to provide theoretical support for rapid detection of pesticide residues in Chinese herbal medicines.

## 1. Introduction

Pesticides are chemicals or biological agents used in agriculture and forestry to prevent and control the growth of pests, including microbes, insects, and weeds, which would alter the growth of desirable plants [1]. Pesticides encompass a wide range of chemicals with broad functions, and their use can greatly improve the yield of crops. However, some pesticides are chemically stable and slow to degrade fully, and they can leave residues on the plant material or in the environment. These residues could then enter the human body through consumption of the plant or via the water or atmosphere. Even small amounts of some residues can impact human health, for example, by inhibiting the immune system or by increasing the risk of cancer [2, 3]. Therefore, it is important to develop tools to sensitively detect pesticide residues in agricultural products to ensure food safety.

Chinese herbal medicines (CHMs) are collected, processed, prepared, and used based on China's traditional medical theory, and most of them are plant-based medicines that have been used for thousands of years [4]. In modern times, pesticides have been widely used in order to reduce damage caused by pests and diseases during cultivation. The presence of pesticide residues on CHMs can endanger human health, but they can also alter the efficacy of medicinal products. Pesticide residues also affect the trade in CHMs: exported CHMs are frequently returned due to contamination, causing economic losses and negatively affecting the virtuous circle of the Chinese medicinal materials' market [5]. Because pesticide residues have such an important and multifaceted impact on CHMs, we have focused our analysis on this particular agricultural product.

In this review, the current situation of pesticide residues in foods and plant-derived CHMs is introduced. We summarize existing detection methods, including traditional chromatographic detection methods and emerging rapid detection methods mostly based on spectroscopy. In particular, we focus on the advantages of the use of surface-enhanced Raman scattering (SERS) in the detection of pesticide residues. We introduce current research regarding the application of SERS technology to pesticide residue detection and summarize challenges that remain in the detection process. Finally, we present prospects of future applications of SERS technology in the detection of pesticide residues in CHMs.

## 2. Methods of Detection of Pesticide Residues in Chinese Herbal Medicines

### 2.1. Present Status of Pesticide Residues in CHMs

The residual pesticides that contaminate plants cultivated for use in CHMs can be classified based on chemical structure into organophosphorus, organochlorine, carbamate, and pyrethroid pesticides. In the cultivation process, contamination of plants with pesticide residues arises from two factors during the growth process: the direct use of pesticides and secondary contamination by pesticide residues in soil and water sources. The rate of detection of pesticides in CHMs has been high in multiple batches and types of materials grown in different regions. For example, a recent study surveyed 152 batches of CHMs, including Maidong samples, Baihe samples, Baishao samples, and Chenpi samples, and the rate of detection of glyphosate residue reached 79.61% [6]. In an analysis of 99 batches of Chuanxiong samples, the rate of detection of dichlorodiphenyltrichloroethane (DDT), chlorobenzamide, and other pesticides was 68.69% [7]. The rate of detection of pyrethroid pesticides in 40 batches of wolfberry samples from different sources was 42.9% [8]. Examples of other pesticide residues that have been found associated with CHMs are shown in Table 1.

### 2.2. Methods of Detecting Pesticide Residues in CHMs

The most important traditional methods of detection are chromatography and chromatography coupled to mass spectrometry (MS), while spectroscopic techniques are usually used for rapid detection. The processes of detection can be generally divided into four steps: collection, extraction, purification, and detection.

#### 2.2.1. Traditional Methods

Gas chromatography-MS (GC-MS) couples the strong separation ability of GC with the accurate identification ability of MS [19]. This technique is associated with high sensitivity and accuracy and is a robust method. Detection limits can be as low as 0.001 *μ*g·kg^−1^, which allows for the trace detection of several types of pesticide residues [20]. However, this technique is unable to measure compounds that are strongly polar, nonvolatile, or thermally unstable. In addition, GC-MS is relatively expensive, as it relies on sophisticated instruments and highly trained professionals.

Liquid chromatography- (LC-) MS can separate thermally unstable and nonvolatile samples and thus can complement the limitations of GC [21]. LC-MS is also associated with highly accurate, sensitive, and reliable results. The detection limit is as low as 0.01 *μ*g·kg^−1^, leading to appropriately qualitative and quantitative capabilities [22, 23]. However, bulky and cumbersome instrumentation has been difficult to adapt to the rapid detection demands of the market.

Thin-layer chromatography uses the adsorption capacity of stationary and mobile phases to achieve the separation of analytes [24]. The method is simple and intuitive, it can analyze multiple samples at the same time, and the detection limit of some pesticides is 0.05 *μ*g·kg^−1^ [25]. In general, this method is associated with low sensitivity, though. Its lack of sufficient separating ability often means that it can only serve as an initial means of separation.

In short, at present, traditional methods of detection are highly accurate and have low detection limits, but the procedures used to perform detection tend to be complicated, expensive, and time consuming. These deficiencies make them difficult to adapt to the current CHM market demands for rapid application and detection.

#### 2.2.2. Spectroscopic Methods

Compared with traditional methods, spectroscopic methods tend to be associated with rapid and simple operation, allowing them to meet the current need for rapid detection technology. Ultraviolet (UV) spectroscopy is based on transitions of valence electrons in target molecules [26]. This technique tends to be simple to operate and requires short detection times. The detection limits of chlorpyrifos and prothioconazole are 4.0 *μ*g·L^−1^ and 0.38 *μ*g·L^−1^, respectively [27, 28]. Nonetheless, it has several deficiencies that greatly limit its application in pesticide residue detection, such as high detection limits and a lack of specificity, and its adaptability needs to be improved.

Near-infrared (NIR) spectroscopy is used to analyze information carried by the substance according to the light transmitted or reflected by an NIR light source [29]. It is associated with fast detection speeds, high efficiency, and low cost, and it can realize the qualitative and quantitative analysis of multiple pesticides, such as profenofos, diazinon, and chlorpyrifos [30–33]. However, at present, the range of application of NIR spectroscopy technology is limited, because it has low sensitivity in the detection of some trace substances, and its ability to detect analytes in liquid samples is insufficient.

Terahertz (THz) spectroscopy is a kind of molecular spectroscopy that is associated with good spectral resolution and strong penetration. It can permit the rapid, nondestructive, and label-free detection of pesticide residues [34]. When combined with chemometrics, qualitative and quantitative analyses of pesticides can be realized, and the detection limit of some pesticides is as low as 0.01 mg·L^−1^ [35, 36]. Unfortunately, THz spectroscopy instrumentation tends to be large and expensive, so portable and rapid detection is unavailable. In addition, analytical results are affected by moisture; the sensitivity and speed of detection thus need to be further improved.

Raman spectroscopy, based on the Raman scattering effect discovered by Indian scientist C. V. Raman, yields spectra associated with inelastic scattering and can be used to interrogate the internal structural information of molecules [37]. The signal arising from chemicals can be very specific and is often referred to as a “fingerprint” [38]. Raman spectroscopy is free from water interference, requires short detection times, and is simple to operate. These advantages have led to its adaptation to the analysis of pesticide molecular structures and the detection of pesticide residues [39]. However, the conventional Raman spectrum signal is very weak, which limits its application in analysis. At present, there are two main ways to enhance the Raman signal: one is the improvement in the laser and the optical path and the other is the use of plasmon-enhanced Raman spectroscopy, which combines nanomaterials with electromagnetic radiation [40].

SERS technology, which combines Raman and nanomaterial technologies, can greatly enhance the Raman signal, leading to the trace detection of pesticide residues. SERS also has characteristics typically associated with more conventional methods, including rapid detection, simple operation, instrumentation portability, and low detection costs. It also has a wide application range and is resistant to interference by off-target molecules.

Currently, SERS is mainly used in the detection of pesticide residues in agricultural products, but there are a few reports regarding its use in detection of pesticide residues in CHMs. For example, SERS was combined with chemometric methods to establish a quantitative prediction model of deltamethrin in *Corydalis yanhusuo*. In this application, gold nanoparticles with a diameter of 75 nm were prepared by a chemical reduction method and then used as a SERS detection enhancement reagent. This technique led to effective predictions and a low detection limit of 0.186 *μ*g·L^−1^ [41]. This was the first application of SERS technology to pesticide residues in CHMs, and it provides a new feasible direction for the rapid detection of pesticide residues.

**Figure 1:**
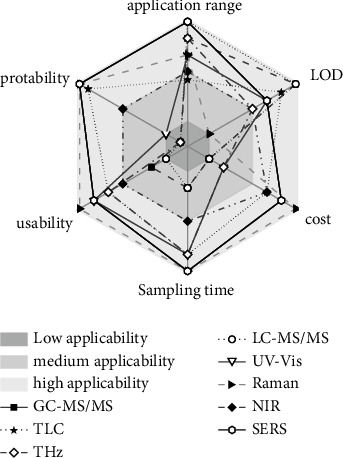
Comparison of the characteristics of different methods used for detecting pesticide residues.

The advantages and disadvantages of various detection methods as applied to the detection of pesticide residues in CHMs are shown in Table 2. To better illustrate the comparison of these detection methods, we developed scores for the application scope, detection limit, cost, sampling time, usability, and portability of a series of detection methods. In this analysis, positive characteristics, including wider application scope, lower detection limit, lower cost, shorter sampling time, and increased usability and portability, are associated with higher scores. As shown in Figure 1, the key strengths of SERS technology are its ability to realize real-time, on-site, and rapid detection of CHMs and its broad application prospects.

## 3. Existing Studies on the Use of SERS in the Detection of Pesticide Residues

**Figure 2:**
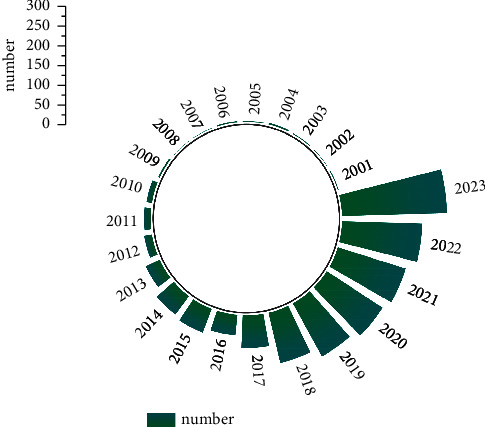
Publication and citation with keywords of sample preparation and SERS from 2001 to 2023 (according to the Web of Science with key words of “Pesticides” and “SERS”).

Recently, several studies have been performed on the use of SERS technology for detecting pesticide residues (Figure 2). Notably, this technology has been used to detect pesticide residues in CHMs, though further research into this application is warranted.

### 3.1. Pretreatment Methods

Sample pretreatment can reduce or even eliminate matrix effects and improve the sensitivity and accuracy of SERS-based detection and analysis. However, it is a time-consuming aspect of the detection of pesticide residues, so developing efficient pretreatment methods is of great significance. Common pretreatment methods include solid-phase extraction and solid-phase microextraction, as well as a modified form of solid-phase extraction called “quick, easy, cheap, effective, rugged, and safe” (QuEChERS) technology. The performance of pretreatment methods is typically evaluated based on recovery rates and other indexes [42, 43].

Solid-phase extraction (SPE) technology is based on solid-liquid chromatographic principles and tends to be relatively simple to implement and to yield highly accurate results [44]. Solid-phase extraction technology has been applied to detect pesticide residues in tea, red bean, orange, and apple. It facilitates the removal of interfering pigments, sugars, proteins, and other impurities, and it is associated with high recovery rates [45–47]. However, traditional solid-phase extraction technology is expensive, has limited enrichment capacity, and produces variable results.

Solid-phase microextraction (SPME) can avoid some shortcomings of solid-phase extraction and is associated with short operation times and less sample consumption [42]. The use of SPME prior to detection of pesticide residues in apples, rice, and wheat has been shown to reduce matrix effects with recovery rates ranging from 79.3% to 106.8% [48–50]. This method is not effective in the treatment of substances with similar polarity or those in complex matrixes.

**Figure 3:**
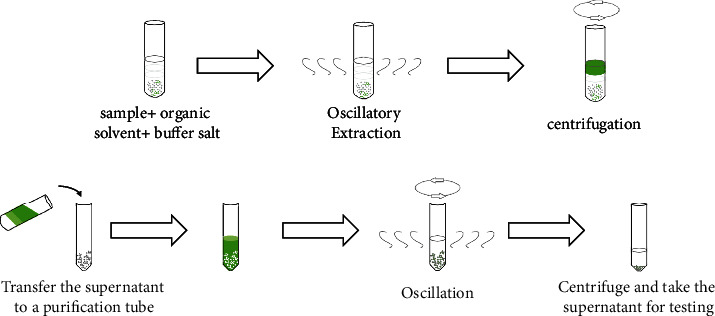
Pretreatment operation process of QuEChERS.

The QuEChERS technique is a simple and rapid pretreatment method related to solid-phase extraction. It minimizes the treatment process, reduces the amount of solvent required, and minimizes environmental pollution (Figure 3) [43]. Using this technique, anhydrous MgSO_4_, primary secondary amine, C18, and graphitized carbon black (GCB) were used as reagents to purify analytes in the detection of chlorpyrifos residues in tea and soil. The pretreatment was found to eliminate interference by impurities such as organic acids and pigments, and the recovery rate was between 93.2% and 103.8% [51, 52]. Interestingly, when Fe_3_O_4_ was used in the pretreatment of agricultural residues in tea, the rate of recovery was better than that when GCB was used [53, 54].

Another substrate that has been used in QuEChERS is multiwalled carbon nanotubes (MWNTs), which have nanoscale hollow tubular structures and large specific surface areas [41, 55]. They have been applied to the detection of triazole residues in vegetables and deltamethrin residues in *C. yanhusuo*. These nanotubes were found to remove pigments, fatty acids, and other interfering substances, and they were associated with high recovery rates.

With its advantages of simplicity and speed, QuEChERS technology has become the preferred pretreatment method for SERS detection of agricultural residues. It showed excellent performance in eliminating the matrix effect of CHMs such as *Codonopsis* and *Astragalus* [8, 56], making it possible for QuEChERS-SERS technology to realize rapid detection of pesticide residues in CHMs. The advantages and disadvantages of several common pretreatment methods as applied to the detection of pesticide residues with SERS are shown in Table 3. It is important to note that some purification materials can adsorb pesticides; therefore, it is necessary to pay attention to the amount of material used during processing.

### 3.2. Nanoreinforced Substrates

**Figure 4:**
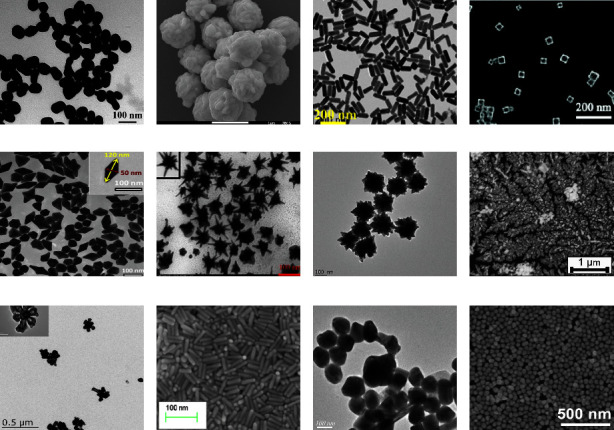
TEM images of different nanosubstrates for SERS analyses: (a) AuNPs; (b) AgNFs; (c) AuNRs; (d) gold nanocages; (e) bipyramid-AuNPs; (f, g) gold nanostars; (h) Ag@Au nanostructures; (i) Au@AgNFs; (j) AuNR array; (k) AuNS@Ag; (l) Au@AgNP array.

There are a wide variety of pesticides, and the residues can be present at low concentrations. Fortunately, the Raman signal can be improved by up to 10 orders of magnitude by the use of high-activity enhanced nanoparticle substrates with the optimized size and shape [57]. SERS substrates include both substrates composed of a single precious metal and those composed of composite materials. TEM images of different nanosubstrates are shown in Figure 4.

**Figure 5:**
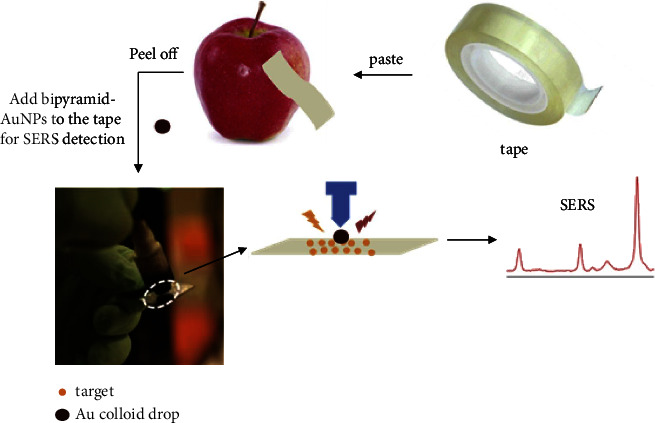
Schematic of the BP-AuNPs-based SERS tape sensor for trace sensing on the fruit peel surface [63].

Single precious metal substrates mainly consist of nanogold or silver. These particles come in different sizes and a variety of shapes, such as spheres, flowers, rods, bipyramids, and stars [58–61]. Gold nanoparticles with an average diameter of 30 nm were used as a reinforcing substrate to detect pesticide residues in Chinese cabbage; here, the limit of detection was 0.5 mg·kg^−1^ [62]. Silver nanoflowers were used to detect the residues of methomyl and acetamiprid in green tea. In this application, voids on the surfaces of the nanoparticles were found to produce local plasmon resonance, which enhanced the Raman signals. In these experiments, the detection limits were 5.58 × 10^−4^ *μ*g·mL^−1^ for methomyl and 1.88 × 10^−4^ *μ*g·mL^−1^ for acetamiprid [44]. When flexible double-cone gold nanoparticles were employed, methylthionine on the surfaces of apples was detected nondestructively with a limit of 31.58 ng·cm^−2^ (Figure 5) [63].

Other highly active SERS composite substrates have been prepared. For example, inert materials and nanomaterials have been used in the assembly of single metal substrates, and amino acids and DNA aptamers have been used as surface modification molecules [64, 65]. The resulting shell-core structure can compensate for shortcomings of raw materials while retaining desirable physical and chemical properties. For example, nanoparticles (NPs) consisting of a silver core and a gold shell (Ag@Au) were found to be stable and to enhance the Raman signal by a factor of 10^7^; using this method led to limits of detection of thiram and thiabendazole on apple peel of 1 mg·kg^−1^ [66].

**Figure 6:**
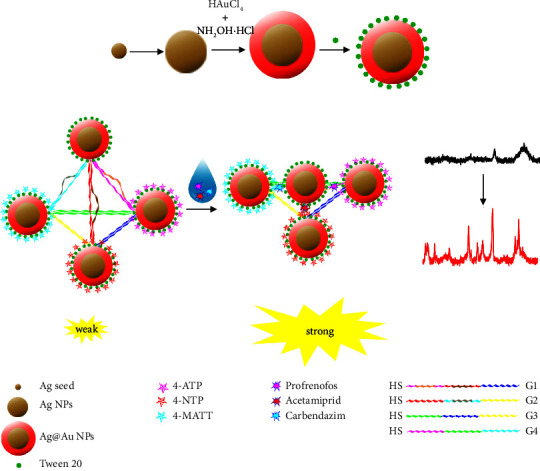
Schematic illustration of the SERS sensor for detecting multiple analytes based on Ag@Au nanotetrahedron [68].

Similarly, cysteamine modification of AuNPs enhances the affinity of acephate to the gold surface, and the resulting detection limit was found to be 0.5 mg·L^−1^ [67]. When a target molecule was linked to a DNA aptamer and then embedded into a nano-tetrahedron based on Au@Ag NPs and a DNA skeleton, as shown in Figure 6, the Raman signal was greatly enhanced, leading to a detection limit as low as 0.0021 ng·mL^−1^ [68, 69].

**Figure 7:**
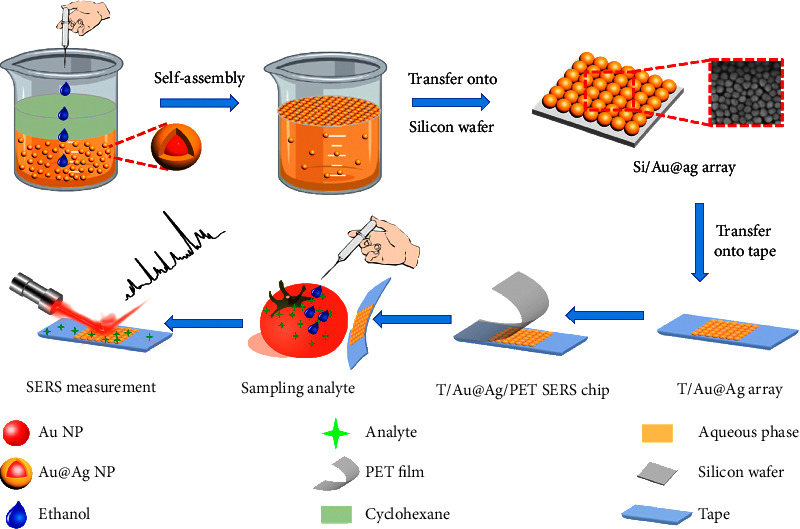
Schematic illustration of the steps for fabricating T/Au@Ag/PET SERS chip and SERS measurement [39].

MOF, SiO_2_, Al_2_O_3_, TiO_2_, Fe_3_O_4_, graphene, molecularly imprinted polymers, and other materials have also been applied to the shells of metal nanoparticles, and these modifications can stably enhance the Raman signal [70–76]. Compared with a Au@Ag substrate, an array of Au@AgNPs had a stronger Raman signal enhancement effect; when they were used to detect thiram on tomato peel with flexible tape, the detection limit was 5 ng·cm^−2^ (Figure 7) [39]. Several enhancement substrates used for pesticide residue detection are shown in Table 4.

Thus, both single precious metal substrates and composite substrates have good SERS enhancement effects. The former are simpler to prepare and have been shown to enhance the Raman signal of pesticide residues, but they tend to be less stable and are difficult to store, so they need to be produced on demand. The latter have better adsorption and affinity and higher stability and sensitivity, whereas they are more complicated to prepare.

### 3.3. Spectral Data Analysis

Prediction models can be established by combining spectral technology with chemometrics. These models involve a rapid spectral analysis that can be completed without sophisticated training, and they can facilitate detection. These processes are accurate and efficient and include spectral preprocessing, model establishment, and model performance evaluation.

An original Raman spectrum contains both chemical information and system disturbance signals. Therefore, it is necessary to preprocess the original spectrum to ensure the accuracy of SERS detection. Common spectral preprocessing methods include the standard normal variate (SNV), multiplicative scatter correction (MSC), mean center (MC), first derivative (D1), and second derivative (D2). When comparing pretreatment performances of SNV, MSC, MC, D1, and D2 in the detection of deltamethrin in wheat, the MC prediction set was found to have the best performance, and the performance following the use of a partial least squares (PLS) model training set was significantly improved [81].

**Figure 8:**
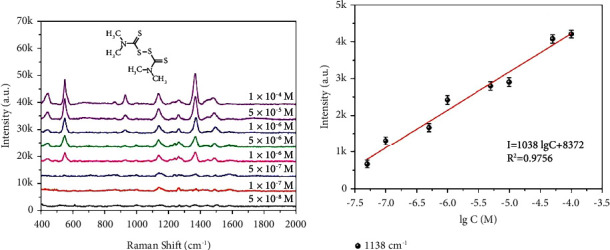
(a) SERS spectra of thiram (from 5 × 10^−8^ M to 1 × 10^−4^ M) adsorbed on the 3D Au@PDMS substrate and (b) the correlation between Raman intensity and concentrations of thiram [82].

Both single-variable and multivariable models can realize quantitative detection of pesticide residues by SERS. A single-variable model is based on the linear equation relating concentration to peak strength. For example, when the residue of thiram on peach peel was detected with a flexible substrate, a linear equation was established with an *R*^2^ of 0.9756, meaning that this model was able to meet the requirements of quantitative detection (Figure 8) [82].

Multivariable modeling methods include PLS, genetic algorithm-partial least squares (GA-PLS), ant colony optimization (ACO), and successive projection algorithm (SPA). The performances of different prediction models are different because models have a variety of spectral intervals and variable selection procedures. At present, the prediction performance of a model is mainly evaluated by the correlation coefficient (*R*^2^), relative percentage difference (RPD), and root mean square error (RMSE), including the root mean square error of calibration (RMSEC) and the root mean square error of prediction (RMSEP). For example, RMSECV and RMSEP have been used as model evaluation indexes, and MC was used as a pretreatment method to compare the performance of PLS, GA-PLS, ACO-PLS, and SPA-PLS. The results showed that MC-SPA-PLS had the best performance and good repeatability in trace detection [81]. Other research on the application of convolutional neural networks and other methods in SERS detection suggest that these methods are efficient in extracting characteristic spectra and lead to improved results [83].

In the actual detection process, a multivariable model can enhance efficiency and accuracy through analyzing the characteristics of different models and selecting an appropriate data processing algorithm according to the scale of required data. However, there is no model database for SERS detection of pesticide residues, and a large amount of preparatory work is required prior to large-scale implementation of this method.

## 4. Conclusion and Prospects

SERS technology has obvious benefits for the rapid detection of pesticide residues as compared with other detection methods. It allows fast detection with high sensitivity, its operation is relatively simple, and its instrumentation is portable. Effective sample pretreatment and flexible substrates are prerequisites for rapid detection. QuEChERS is the preferred pretreatment method for SERS detection of agricultural residues, because it is simple and fast, can greatly reduce the pretreatment time, and can remove matrix effects to the greatest extent while ensuring a high recovery rate. Flexible enhanced substrates have the advantages of simple preparation, good stability, and strong Raman signal enhancement effects, and they can permit the realization of nondestructive detection of trace contaminants. As shown in Table 4, SERS is presently able to achieve trace detection or even ultratrace detection, and the detection limits are lower than relevant provisions of the maximum residue limit of pesticides in the current national standard for food safety. It is likely, then, that SERS technology can provide conditions for rapid detection of pesticide residues in CHMs. Besides, combining spectral technology with chemometrics can establish prediction models, and the accuracy of quantitative detection can be greatly improved.

However, some deficiencies remain in the application of SERS technology for detecting pesticide residues in CHMs. First, CHMs have complex chemical compositions, and some physical and chemical properties are similar to those of pesticide components, which makes extraction, analysis, and detection more difficult. Second, the complicated chemical composition of CHMs means that matrix effects are more complex; importantly, SERS-based detection is associated with an amplified matrix effect, which affects the accuracy of detection results. Third, there are no specific standards available for pesticide residues in the detection of CHMs by SERS, which hinders the implementation of SERS technology.

Research into the detection of pesticide residues in CHMs by SERS should take several forms: (1) optimization of enhanced substrates by changing the size and morphology to develop materials with good reinforcement effects and high stability and to allow additional control over the amount of reactants, reaction temperature, reaction time, and stirring; (2) optimization of pretreatment methods: simple and fast pretreatment methods should be chosen according to the properties of the tested substance to ensure high recovery rates and accuracy; and (3) optimization of algorithm models and the establishment of SERS pesticide residue detection databases, as well as acceleration of the establishment of SERS pesticide residue detection standards for CHMs. With this research progress, it is believed that SERS technology will begin to have a broader market appeal, and it will be possible to realize rapid and real-time detection of pesticide residues in CHMs.

## Figures and Tables

**Table 1:** Pesticide residues associated with some Chinese herbal medicines.

Samples	Medicinal part	Analytical techniques	Types of pesticide residues	Ref.
*Panax ginseng*	Rhizome	GC-MS/MS	Acetochlor, chlorpyrifos, procymidone, triadimefon, propiconazole	[9]
*Glycyrrhiza uralensis*	Rhizome	GC-MS/MS	*β*-Endosulfan, thiosulphate, deltamethrin	[10]
*Lycopus lucidus*	Rhizome	GC-MS/MS	Permethrin, propiconazole, benalaxyl, isazofos	[11]
*Codonopsis radix*	Rhizome	GC-MS/MS	Chlorpyrifos, acetochlor, propiconazole	[12]
Chuanxiong rhizoma	Rhizome	LC-MS/MS	Triazophos, carbofuran, DDT, carbendazim, dimethomorph	[7]
*Angelica sinensis radix*	Rhizome	GC-MS/MS	Organochlorines, pyrethroids, dinitroanilines	[13]
*Panax notoginseng*	Root	GC-MS/MS	Dichlorvos, sulfotep	[14]
*Fritillaria*	Bulb	LC-MS/MS	Carbendazim, permethrin, chlorpyrifos, acetochlor	[15]
*Dendrobium officinale*	Rhizome	GC	Pyrethroids	[16]
Honeysuckle	Flower	GC-ECD	Cyfluthrin, omethoate, triazophos	[17]
Wolfberry	Fruit	HPLC-MS/MS	Cyfluthrin, fenvalerate, carbendazim, methomyl	[18]

**Table 2:** Comparison of advantages and disadvantages of methods used to detect pesticide residues.

Detection methods	Advantages	Disadvantages
GC-MS/MS	Good sensitivity, accuracy and precision, high analysis efficiency, and wide application range	Unsuitable for analysis of compounds that are strongly polar, nonvolatile, or thermally unstable; expensive instrumentation
LC-MS/MS	Wide analysis range; able to analyze compounds that GC-MS/MS cannot	Complicated, cumbersome, and expensive instrumentation
TLC	Rapid detection and low cost; simple and portable instrumentation; strong selectivity	Low sensitivity and poor separation ability
UV-Vis	High sensitivity, simple operation; can simultaneously analyze multiple compounds	Potential for spectral interference caused by overlapping spectral lines; relatively low selectivity
NIR	Wide application range, can provide structural information	Not suitable for analyzing water-containing samples; data analysis is complex
THz	Rapid and nondestructive detection	Instrumentation is cumbersome and expensive; low detection sensitivity
Raman	Rapid detection, simple and portable instrumentation; provides “fingerprints” of target substances	Weak spectral signal; poor sensitivity and precision
SERS	Fast detection; portable instrumentation with simple operation; high sensitivity	Easily disturbed by external factors; low stability of quantitative calculation models

**Table 3:** Comparison of advantages and disadvantages of pretreatment methods.

Pretreatment methods	Advantages	Disadvantages
SPE	Sample enrichment and purification can be completed at the same time, simple operation	High cost, limited enrichment capacity, poor repeatability
SPME	No need for organic solvents, low environmental pollution; simple instrumentation and easy to operate	Poor repeatability, low accuracy of quantitative detection, few types of commercially available polymers
QuEChERS	Rapid and simple operation, good sensitivity and accuracy, wide application range, less environment pollution	Impurities can be only partially removed, susceptible to matrix effects

**Table 4:** Pesticide residues detected by SERS.

Samples	Pesticide	SERS substrate	Detection limit	Maximum residue limit	Ref.
Chinese cabbage	Acetamiprid	AuNPs	1 mg·kg^−1^	1 mg·kg^−1^	[62]
	Malathion		1 mg·kg^−1^	8 mg·kg^−1^	
	Phosmet		0.5 mg·kg^−1^	0.5 mg·kg^−1^	

Apple	Triazophos	AgNPs	0.02 mg·kg^−1^	0.2 mg·kg^−1^	[77]

Green tea	Methomyl	AgNFs	0.6 *μ*g·L^−1^	0.2 mg·kg^−1^	[44]
	Acetamiprid		0.2 *μ*g·L^−1^	0.5 mg·kg^−1^	

Tomato/apple peel	Methylthionine	Bipyramid-AuNPs	31.58 ng·cm^−2^	—	[63]

Tea	Paraquat	Gold nanostars	0.2 mg·kg^−1^	0.5 mg·kg^−1^	[49]

Peach	Thiacloprid Profenofos	Au@AgNPs	0.1 mg·kg^−1^	0.5 mg·kg^−1^	[78]
			0.01 mg·kg^−1^	0.05 mg·kg^−1^	

Apple	Thiram	Ag@AuNPs	1 mg·kg^−1^	5 mg·kg^−1^	[66]
	Thiabendazole		1 mg·kg^−1^	15 mg·kg^−1^	

Rice	Acephate	AuNRs with cysteine	0.5 mg·L^−1^	1 mg·kg^−1^	[67]

Tomato peel	Thiram	Au@AgNP array	5 ng·cm^−2^	5 mg·kg^−1^	[39]

Cucumber	Acetamiprid	Ag@SiO2	2.66 ng·mL^−1^	1 mg·kg^−1^	[71]

Tomato/grape peel	Chlorpyrifos	Ag/TiO2 nanorods	2 ng·cm^−2^	0.5 mg·kg^−1^	[73]
			5 ng·cm^−2^	0.02 mg·kg^−1^	

Apple peel	Thiram	AuNR array	0.41 ng·cm^−2^	5 mg·kg^−1^	[79]

Centella	Chlorpyrifos	GO-Au nanocomposites	0.1 mg·kg-1	0.5 mg·kg-1	[80]

## Data Availability

Data availability is not applicable to this article as no new data were created or analyzed in this study.
